# Membrane Transporters as Mediators of Cisplatin Effects and Side Effects

**DOI:** 10.6064/2012/473829

**Published:** 2012-11-25

**Authors:** Giuliano Ciarimboli

**Affiliations:** Experimentelle Nephrologie, Medizinische Klinik D, Universitätsklinikum Münster, Albert-Schweitzer-Campus 1, Gebäude A14, 48149 Münster, Germany

## Abstract

Transporters are important mediators of specific cellular uptake and thus, not only for effects, but also for side effects, metabolism, and excretion of many drugs such as cisplatin. Cisplatin is a potent cytostatic drug, whose use is limited by its severe acute and chronic nephro-, oto-, and peripheral neurotoxicity. For this reason, other platinum derivatives, such as carboplatin and oxaliplatin, with less toxicity but still with antitumoral action have been developed. Several transporters, which are expressed on the cell membranes, have been associated with cisplatin transport across the plasma membrane and across the cell: the copper transporter 1 (Ctr1), the copper transporter 2 (Ctr2), the P-type copper-transporting ATPases ATP7A and ATP7B, the organic cation transporter 2 (OCT2), and the multidrug extrusion transporter 1 (MATE1). Some of these transporters are also able to accept other platinum derivatives as substrate. Since membrane transporters display a specific tissue distribution, they can be important molecules that mediate the entry of platinum derivatives in target and also nontarget cells possibly mediating specific effects and side effects of the chemotherapeutic drug. This paper summarizes the literature on toxicities of cisplatin compared to that of carboplatin and oxaliplatin and the interaction of these platinum derivatives with membrane transporters.

## 1. Introduction

A general concept of drug movement across biological membranes is that they can pass cell membranes via passive diffusion at a rate related to their lipophilicity. However, it is becoming evident that membrane transporters are also important determinants of in vivo drug disposition, therapeutic efficacy, and adverse drug reactions [1]. Many membrane transporters have a specific tissue and also cell distribution. In epithelial tissues, which are constituted by polarized cells, transporters are even specifically expressed on the apical or basolateral cell membrane. In this way, a specific drug-transporter interaction can be used to target drugs to selected cells and tissues but can also induce specific undesired adverse effects [2]. Carrier-mediated cellular drug accumulation is a resultant of the activity of uptake and efflux transporters. The pharmacological significance of efflux transport proteins is evident considering their role in the development of resistance of tumor cells to chemotherapeutic agents (as, e.g., in the case of P-glycoprotein [3]) or in the induction of drug cellular toxicity because of their malfunction (as, e.g., in the case of multidrug resistance-associated protein 2 polymorphism [4]). The pharmacological and more specifically the toxicological role of uptake transporters in the development of specific drug adverse effects has been only in the recent years under critical investigation. Transporter-mediated uptake has been shown to be an important process mediating cellular accumulation of cisplatin (for review, see [2, 5–7]). Cisplatin is an important chemotherapeutic drug used in the therapy of a broad spectrum of human malignancies such as ovarian, testicular, head and neck, and lung cancer. In the early 1970s, metastatic testicular cancer was associated with only 5% survival. Today, with the use of surgery techniques together with modern chemotherapy, based on combination of cisplatin with bleomycin and etoposide, testicular cancer has become a model for a curable neoplasm [8], underlining the importance of cisplatin in tumor therapy. The action of cisplatin on cell growth was unexpectedly discovered by Rosenberg in 1965 by investigating the effects of an electric field on the growth of *Escherichia coli bacteria* [9]. When placed in an electric field using platinum-conducting plates, bacteria ceased to divide. Rosenberg hypothesized that if cisplatin could inhibit bacterial cell division it could also suppress tumor cell growth. Cisplatin was approved by the FDA in 1978 for the treatment of metastatic testicular or ovarian cancer and is also administered for many other types of solid tumors. Cisplatin is one of the most widely utilized antitumor drugs in the world, with annual sales of approximately $500 million (US) [10]. The treatment with cisplatin is associated with dose-limiting side effects such as nephrotoxicity, ototoxicity, and peripheral neurotoxicity [11]. For this reason, numerous platinum derivatives have been further developed with more or less success to minimize toxic effects. Carboplatin was approved in March 1989 for treatment of ovarian cancer, and in 1994 a third-generation platinum drug, oxaliplatin, was approved for treatment of metastatic colorectal cancer. Cisplatin is still used regularly for head and neck and germ cell tumors, while carboplatin has replaced cisplatin for most ovarian tumors and for the treatment of non-small-cell lung carcinoma [11, 12]. Oxaliplatin is currently approved for treatment of colorectal cancer but has also been shown to have activity against breast and endometrial cancers and malignant melanoma in phase I studies (reviewed in [13]). Additional phase II trials show oxaliplatin to be active against non-small-cell lung cancer, prostate cancer, germ-cell malignancies, ovarian carcinoma, non-Hodgkin's lymphoma, and malignant mesothelioma; minimal or no activity was observed in head and neck carcinoma and in malignant astrocytoma [11, 13]. Even though several other platinum derivatives have been developed and used in the therapy of cancer, the focus of this work is on cisplatin, its toxicities, and its cellular transport as process mediating effects and side effects in comparison with that of carboplatin and oxaliplatin.

A common event happening when platinating agents enter a cell is their aquation that is losing of chloride or oxalate ions and gaining two water molecules to form aquaions. The low intracellular concentration of chloride ions facilitates this process. The positively charged aquated form is more reactive to the cellular targets, such as nucleophilic molecules within the cell, including DNA, RNA, and proteins [11, 14]. It is generally accepted that DNA is the preferential cytotoxic target for cisplatin and other platinating agents: these substances bind preferentially the imidazole ring of the purines guanosine and adenosine forming monoadducts, intrastrand crosslinks, and interstrand crosslinks [11]. All crosslinks distort the structure of the DNA duplex and begin the DNA damage response signaling, resulting in cell cycle arrest and apoptosis (reviewed in [15]). Cisplatin and carboplatin intrastrand crosslinks bend the double helix by 32–35° toward the major groove, whereas oxaliplatin treatment bends the helix even further [11]. Oxaliplatin forms fewer crosslinks than cisplatin at equimolar concentrations; however, it is able to induce similar numbers of single-strand and double-strand breaks on DNA. Moreover, oxaliplatin adducts are bulkier and more hydrophobic than those formed from cisplatin or carboplatin, leading to different effects in the cell (reviewed in [11]). Wang and Lippard [14] underlined that the cisplatin and oxaliplatin adducts on DNA are differentially recognized by mismatch repair proteins and some damage-recognition proteins, such as high-mobility group box protein 1, TATA box-binding protein, and human upstream binding factor. Moreover, DNA polymerase *η* has a greater efficiency of error-free bypass of oxaliplatin adducts compared with cisplatin [14]. These disparate recognition and processing events are thought to contribute to differences in cytotoxicity and to the range of anticancer activity shown by oxaliplatin and cisplatin [14].

## 2. Cisplatin Toxicities

This paragraph focusses on the main side effects of cisplatin treatment (nephrotoxicity, ototoxicity, and neurotoxicity) in comparison with carboplatin and oxaliplatin.

### 2.1. Nephrotoxicity

In patients cisplatin-induced nephrotoxicity manifests acutely and/or chronically. A decrease in renal plasma flow was observed very early in patients receiving cisplatin at a dose of 20 mg/m^2^ over a period of 4 h, and an increase in urinary enzymes occurred rapidly [16]. In these patients hypomagnesaemia, hypocalcaemia, and hypokalemia were frequently observed [16]. Comparing the long-term effects of cisplatin administration at low (20 mg cisplatin/m^2^) and high (40 mg/m^2^) doses after 4 cycles of daily treatment for 5 days, a small but significant decrease in ^51^Cr-EDTA clearance was observed in the low-dose group [17]. In the high-dose cisplatin group, a severe progressive decrease in glomerular filtration rate (GFR) was observed during treatment and GFR remained decreased for up to 2 years after termination of treatment [17]. The observation of an acute increase in N-acetyl-beta-D-glucosaminidase and beta-2-microglobulin indicated a primary tubular effect of cisplatin in humans. A marked reduction of proximal tubular reabsorptive capacities of sodium and water was also observed in the high-dose group, together with a decrease in distal tubular function. These changes persist for at least 6 months after treatment. In the high-dose group proteinuria developed [17]. This was mainly of tubular origin during cisplatin infusion and of glomerular origin between treatment cycles [17].

Clinically, cisplatin nephrotoxicity develops after 10 days of cisplatin administration and is manifested as lower glomerular filtration rate, higher serum creatinine, and reduced serum magnesium and potassium levels [18]. Interestingly, striking differences between patients in susceptibility to progressive nephrotoxicity are observed [19]. Even though nephrotoxicity can be controlled by diuretics and prehydration of patients, it is recognized that the prevalence of cisplatin nephrotoxicity is high, occurring in about one-third of patients undergoing cisplatin treatment [18]. In animal studies it has been shown that the kidney accumulates more cisplatin than other organs [20] and that the proximal tubules are principally damaged by cisplatin [21]. Cisplatin-induced nephrotoxicity is initiated by an acute, mainly proximal tubular impairment, preceding alterations in renal hemodynamics. Performing micropuncture experiments in rat kidney, it was demonstrated that at 48 to 72 hours after administration of cisplatin depressed renal function is due to impairment of proximal as well as distal tubular reabsorptive capacities associated with increased renal vascular resistance [17]. In murine models of cisplatin-induced acute renal failure it has been shown that, after cisplatin administration, cells of the S3 segment in the renal proximal tubule are especially sensitive and undergo extensive necrosis in vivo. Similarly, cultured proximal tubule cells undergo apoptosis in vitro after cisplatin exposure [22]. Using cultured renal tubular cells, it was suggested [23] that the dosage of cisplatin might determine whether the cells die by necrosis or apoptosis. Necrotic cell death was observed when a high concentration of cisplatin (millimolar) was used, while lower concentrations of cisplatin (micromolar) led to apoptosis [23]. In experimental models and in patients, carboplatin treatment rarely results in nephrotoxicity [11, 24, 25]. Nephrotoxicity has not been reported in any of the oxaliplatin trials, allowing administration of oxaliplatin without hydration [26].

### 2.2. Ototoxicity

Ototoxicity is an untypical side effect for a chemotherapeutic drug. Cisplatin treatment causes a hearing loss, which can also lead to deafness. Ototoxicity remains an unresolved clinical problem especially in infants and younger children, where it leads to a considerable risk of delayed language development due to impaired perception of higher frequency consonant sounds that is of great importance in the presence of background noise. This may have devastating consequences for a young child's social and educational development [27]. The incidence of ototoxicity is reported to be between 23 and 50% in adults and greater than 50% in children [11]. The clinical symptoms of toxicity consist of bilateral symmetrical high-frequency sensorineural hearing loss, ear pain, or tinnitus [28]. When a child has communicatively significant hearing loss, technology to improve hearing is beneficial. Hearing aids do not restore hearing but provide acoustic access to speech and sounds. FM systems (devices that transmit sounds over FM radio waves) reduce the negative effects of distance, reverberation, and background noise in the school setting.

Cisplatin has been shown to target three areas in the cochlea: the hair cells in the basal turn of organ of Corti, the spiral ganglion cells, and the lateral wall tissues (spiral ligament and stria vascularis). After treatment with cisplatin outer hair cells, cells in the stria vascularis, and spiral ligament each has been shown to undergo apoptosis [29]; in nuclei of these cells platinated DNA immunoreactivity has been detected [29].

Damage induced by cisplatin begins at the cochlea base, where high-frequency sounds are processed, and proceeds towards the apex, affecting also hearing at lower frequencies as the cumulative dose increases [30]. In the cochlea, cisplatin seems to induce the generation of reactive oxygen species and/or the depletion of scavenging enzymes causing cell apoptosis [29].

Carboplatin seems to be much less ototoxic than cisplatin [31], and clinically significant deafness does not occur with conventional dosing making routine audiometric monitoring unnecessary [32]. Oxaliplatin is rarely ototoxic. Since in guinea pig cochlea total platinum concentration and perilymphatic drug concentrations were lower after intravenous oxaliplatin treatment (16.6 mg/kg) than after equimolar cisplatin treatment (12.5 mg/kg), it has been proposed that lower cochlear uptake of oxaliplatin than cisplatin appears to be a major explanation for its lower ototoxicity [33].

### 2.3. Neurotoxicity

Most patients treated with cisplatin develop a symptomatic and clinically detectable sensory neuropathy, caused by its preferential uptake in the dorsal root ganglia, which produces a dose-related large fibre sensory neuropathy (neuronopathy) [34, 35]. Symptoms include unpleasant distal paresthesias (tingling in the extremities) and numbness, associated with large fibre sensory loss (reduced vibration and joint position sensations) and diminished or absent muscle stretch reflexes [34, 36–38]. Sensory ataxia (incoordination) may be disabling in those patients who have severe neuropathy. These symptoms may appear as soon as one month after initiating treatment [34]. The neuropathy may only partially recover or not recover at all. In rodents, cisplatin affects sensory nerve structure and function, showing preferential toxicity to large diameter neurons and proprioceptive sensory modalities, while motor nerves are spared from toxicity (reviewed in [39]). In this way, the pattern of cisplatin-induced peripheral neurotoxicity in rodents is similar to that observed in patients [39]. The pharmacokinetics of cisplatin and its concentration in the peripheral nervous system resulting from treatment are comparable in rodents and humans [39].

The mechanism of platinum neurotoxicity remains incompletely understood although it may involve platinum accumulation within the dorsal root ganglia (DRG) leading to atrophy or loss of peripheral sensory neurons [40]. Clinical and electrophysiological features of the sensory neuropathy and the sparing of motor function point to damage occurring at the level of the cell body of sensory neurons within the DRG [36, 37, 40–42]. Histopathological studies have shown altered size profiles of DRG neurons after platinum treatment of patients [36, 37, 40, 42, 43] and in rodent models [40, 44–50], consistent with induction of neuronal atrophy or selective loss of large DRG neurons. High levels of platinum accumulate in the DRG compared to peripheral nerves, spinal cord, and brain, following exposure to platinum-based drugs in patients [36, 43, 51] and animal models [45, 46, 52–54], but the differing neurotoxicity profiles of cisplatin, carboplatin, and oxaliplatin are not simply explained by differences in DRG platinum concentration [47, 52, 53]. DRG appears to express some transporters involved in the uptake of platinum derivatives (see the following), such as copper transporter 1 [55], electroneutral organic cation transporters 1 and 2 (OCTN1, and OCTN2) [56]. However, these transporters are also expressed in the brain neurons [57–59]. At my knowledge, there is no information on the expression of multidrug and toxin extrusion protein 1 and of organic cation transporters in DRGs.

Carboplatin is considered to be less neurotoxic than cisplatin [60]. Neurological dysfunction is a side effect in the carboplatin-based regimen but appears later on and mostly after the administration of carboplatin at high-dose levels or in combination with other cytotoxic agents known to be neurotoxic (e.g., taxanes) (reviewed in [60]). Only 4%–6% of patients who receive carboplatin may develop peripheral neuropathy [60].

Conversely, oxaliplatin treatment is associated with the development of significant neurological dysfunction. As in the case of cisplatin, peripheral neuropathy is the most common dose-limiting toxicity of oxaliplatin, and it is one of the major causes of discontinuation of therapy [60]. However, two forms of sensory peripheral neuropathy caused by administration of oxaliplatin can be distinguished: an acute peripheral sensory neuropathy may appear during the administration of the drug or after the first few drug infusions and a chronic dose-limiting cumulative peripheral sensory neuropathy [60]. The mechanisms underlying these two forms of oxaliplatin-induced peripheral neuropathy have not been clearly defined. Acute neurotoxic effects may result from the impairment of voltage-gated sodium channels and occur approximately in 85%–95% of all patients exposed to oxaliplatin [60–62]. From a mechanistic point of view, the inhibition of neurite outgrowth but not tumor cell death induced by oxaliplatin seems to be associated with reductions in intracellular calcium and of growth-associated protein-43 expression. Interestingly, this inhibition was suppressed by the addition of a combination of calcium gluconate and magnesium sulfate. This combination may be useful for protecting against oxaliplatin-induced neurotoxicity without reducing the antitumor activity of oxaliplatin [63]. Another hypothesis that may explain this side effect is the induction of oxidative stress, which can be an important pathogenetic mechanism and, possibly, a therapeutic target to limit the severity of platinum-induced peripheral neurotoxicity but preserving the anticancer effectiveness [64]. Symptoms consist mainly of paresthesias and dysesthesias in the extremities and the perioral region and are exacerbated by cold exposure [60].

The most accepted mechanism for the chronic form of oxaliplatin-induced neurotoxicity is decreased cellular metabolism and axon plasmatic transport resulting from the accumulation of oxaliplatin in the dorsal root ganglia cells. As a result, oxaliplatin produces symmetrical, axonal, and sensory distal neuropathy [60, 65]. Neurological symptoms in this form of sensory neurotoxicity are dominated by pronounced paresthesias and dysesthesias of the extremities and dysfunction of fine sensory-motor coordination which may result in impairment of daily life [12, 60, 66]. The incidence of chronic oxaliplatin-induced peripheral neuropathy is related to various risk factors such as cumulative dose, treatment schedule, and time of perfusion [60, 67].

## 3. Cellular Transport of Cisplatin

The first step for cisplatin to exert its toxic effects is to enter the cells. The uptake route of cisplatin across plasma membrane is not completely understood. Originally, a passive diffusion across the plasma membrane has been proposed as process responsible for cisplatin transport into the cell [68]. Such a process is unspecific and could hardly contribute to explain the specific toxicity of cisplatin. In the last years, lines of evidence accumulated that the cellular uptake of cisplatin is mediated, at least in part, by transport proteins. Several transporters, which are expressed on the cell membranes, have been associated with cisplatin transport across the plasma membrane and across the cell: the copper transporter 1 (Ctr1), the copper transporter 2 (Ctr2), the P-type copper-transporting ATPases ATP7A and ATP7B, the organic cation transporter 2 (OCT2), and the multidrug extrusion transporter 1 (MATE1). Some of these transporters are also able to accept other platinum derivatives as substrates. Since membrane transporters display a specific tissue distribution, they can be important molecules that mediate the entry of platinum derivatives in target and also non target cells possibly mediating specific effects and side effects of the chemotherapeutic drug. In the next paragraph these transporters and their interaction with cisplatin, carboplatin, and oxaliplatin will be described.

### 3.1. Copper Transporter 1 (Ctr1)

According to the Human Genome Organisation (HUGO), human transporters are classified based on their amino acid sequence in 43 solute carrier (SLC) families [69]. Copper transporters have been assigned to the SLC31A family. Copper is an essential nutrient for almost all eukaryotic organisms to carry out biological processes such as free radical detoxification, mitochondrial respiration, iron metabolism, neuropeptide maturation, connective-tissue formation, and pigmentation [70]. Therefore, it is evident that transport systems for copper are necessary for every cell. Indeed, Ctr1 (SLC31A1) is ubiquitously expressed in tissues and is particularly abundant in the choroid plexus, renal tubules, and in connective tissues of the eye, ovary, and testis [71]. The levels of Ctr1 in tissues may be influenced by the physiological state, such as pregnancy or lactation [72]. Ctr1 is a 23-kDa channel-like transporter, which has a unique pore for the transport of copper, three transmembrane domains, and oligomerizes to form a functional trimer [70, 73, 74]. Ctr1 mediates energy-independent copper transport with an apparent *K*_*m*_ of 2–5 *μ*M [75]. The transport of copper by Ctr1 is strongly inhibited by silver and not by divalent cations, suggesting that the reduced form of copper, Cu(I), is substrate of the transporter [75]. Global genetic deletion of Ctr1 is embryonic lethal [71, 74], probably due to insufficient copper supply to the body. However, a function of Ctr1 independent from copper in early tissue morphogenesis can be also speculated [76]. Yonezawa and Inui [5] recently underlined that Ctr1 is an equilibrative transporter. This fact could be important in the comparison with the OCT2, a concentrative transporter (see the following). Ctr1 has been demonstrated to mediate the cellular uptake of cisplatin [77–80]. Interestingly, it has been reported that in both yeast and mouse embryonic fibroblasts, knockdown of Ctr1 reduces cisplatin uptake by almost 80% [81, 82]. Moreover, various cell lines overexpressing Ctr1 accumulate significantly high levels of cisplatin [77].

Using immunohistochemistry, Ctr1 was found to localize to the apical membrane in intestinal epithelial cells of the mouse, rat, and pig [83]. Ctr1 has been shown to be highly expressed in the mouse kidney [72, 84], apparently on the basolateral side of proximal tubules cells [85]. In vitro experiments with human embryonic kidney (HEK293) cells transfected with Ctr1-specific siRNA showed a significantly reduced cisplatin uptake compared to cells transfected with control siRNA [85]. However, cimetidine, a substrate of organic cation transporters (OCTs), had also partially inhibitory effects on cisplatin uptake in HEK293 cells. Importantly, it was also shown that cimetidine could further reduce cisplatin uptake in Ctr1 knockdown HEK293 cells. The additive effects of cimetidine and Ctr1 knockdown were also shown for the toxicity of cisplatin: both apoptosis and necrosis induced by cisplatin were further reduced by cimetidine in Ctr1 knockdown HEK293 cells [85]. According to these results, it could be hypothesized that not only Ctr1 but also a cimetidine-inhibitable transport system, probably an organic cation transporter, contribute to cisplatin transport in renal tubular cells, resulting in nephrotoxicity. However, it must be considered that cimetidine may have off-target effects.

Ctr1 has been identified in the mouse cochlea, where it is expressed in outer hair cells (OHCs), inner hair cells (IHCs), stria vascularis, spiral ganglia, and surrounding nerves [86]. In a further study, Ctr1 was localized in the organ of Corti, in the epithelium of the stria vascularis, and in the spiral ganglion neurons of postnatal day 3 rat cochlear organotypic cultures [87]. Interestingly, these structures represent the three major targets of cisplatin-induced cochlear damage [88]. Blocking Ctr1 by copper sulfate was suggested to be effective in protecting hair cells against cisplatin toxicity [86]. However, further studies indicated only a modest protective action of hair cells by coadministration of copper sulfate [87]. Furthermore, it has been observed that copper sulfate per se can be toxic for these cells [87]. Of interest is the observation that also, in this case, coadministration of cimetidine provided considerable protection against cisplatin-induced hair cell loss in cochlear cultures, suggesting the existence of a cimetidine-sensitive cisplatin uptake route also in these cells.

For what concerns peripheral neurotoxicity, there is little information regarding the presence of transporters in dorsal root ganglia. The Ctr1-mRNA has been identified in rat dorsal root ganglia (DRG) [40]. Ctr1 immunoreactivity was associated with the cell bodies of DRG neurons but not with their nerve fibers or other tissue elements of the DRG [40]. It was localized to the neuronal surface, with a plasma membrane pattern of immunoreactivity along with staining of cytoplasmic vesicular structures. Interestingly, the expression of Ctr1 protein was evident in a subpopulation of larger-sized DRG neurons that underwent atrophy in response to cisplatin and oxaliplatin treatment. Conversely, the smaller Ctr1-negative DRG neurons were less atrophic after treatment with oxaliplatin or cisplatin than the CTR1-positive neurons. Indeed, some studies suggested that large-sized DRG neurons are more vulnerable to damage from platinum drug treatment than small-sized DRG neurons [49, 50] and that peripheral neurotoxicity is caused by neuronal atrophy [36, 43, 44, 46, 48–50]. Comparing the amount of cell body atrophy of Ctr1-expressing DRG neurons induced by the treatment of rats with equitoxic doses of cisplatin, oxaliplatin, and carboplatin, the authors found a ranking of these platinum agents according to their effect on the size profiles of Ctr1-immunoreactive DRG neurons with oxaliplatin having the greatest effect, followed by cisplatin and then carboplatin [40]. This ranking corresponds with the relative cumulative dose potencies of oxaliplatin, cisplatin, and carboplatin for reducing sensory nerve conduction velocity in rats [53].

Ctr1 seems to control the cellular accumulation of cisplatin, carboplatin, and oxaliplatin at low concentrations. However, at higher concentrations, cellular accumulation of oxaliplatin is not dependent on Ctr1 [79].

A discrete expression of Ctr1-mRNA was detected in several cell lines from human tumor samples [89], suggesting that this transporter could represent the uptake route of cisplatin in cancer cells.

Indeed, there are some clinical studies suggesting the importance of Ctr1 for cisplatin tumor resistance. Twenty-two single nucleotide polymorphisms (SNP) of Ctr1 have been identified by the screening of 282 non-small-cell lung carcinoma (NSCLC) Chinese patients. A significant relationship was found between rs7851395 and rs12686377 polymorphisms and platinum resistance, as well as clinical outcomes [90].

The expression levels of Ctr1 may be important to determine cell sensitivity to cisplatin. Indeed, using three pairs of cisplatin-resistant cell lines and two ovarian cancer cell lines derived from patients, where the platinum-based chemotherapy failed, it was demonstrated that the resistance against cisplatin is associated with reduced expression of the hCtr1 [91]. Interestingly, the cells become again sensitive for cisplatin by treatment with copper-lowering agents, which are able to enhance hCtr1 expression. Such a preferential induction of hCtr1 expression in cisplatin-resistant variants by chelation of copper can be explained by activation of mechanisms, which regulate copper homeostasis [91]. Enhanced cell-killing efficacy by a copper-lowering agent was also observed in animal xenografts bearing cisplatin-resistant cells [91]. Finally, by analyzing a public gene expression dataset, the same authors found that ovarian cancer patients with elevated levels of hCtr1 in their tumors, but not ATP7A and ATP7B, had more favorable outcomes after treatment with platinating drugs than those expressing low hCtr1 levels [91]. Other lines of evidence of the importance of hCtr1 expression for efficacy of treatment with platinating agents derive from the study of the Ctr1-mRNA expression in 40 women with ovarian carcinoma: high Ctr1 expression was significantly associated with sensitivity to platinum-based chemotherapy and progression-free survival [92]. Conversely, low Ctr1 expression was significantly associated with resistance to platinum-based chemotherapy and the shortest survival [92].

Taken together these results suggest that Ctr1 is important for the cellular uptake of cisplatin, carboplatin, and oxaliplatin in tumor cells. However, an additional transport system appears to be present and to mediate specific uptake of cisplatin in nontarget tissues, such as renal and cochlear tissue.

### 3.2. Copper Transporter 2

(Ctr2, SLC31A2) is a copper transport protein with substantial structural homology to Ctr1 [93]. In mammalian cells, Ctr2 seems to be also involved in the transport of copper: in African green monkey kidney COS-7 cells Ctr2 promoted the uptake of copper [94]. However, its cellular distribution appears to be slightly different from that of Ctr1: Ctr2 is not only localized in the plasma membrane [94, 95], but also in late endosomes and lysosomes [93]. The main function of Ctr2 seems to reside in mediating the efflux of copper from endosomes and lysosomes under conditions of low environmental copper concentration [95]. A similar function was proposed for cisplatin [96]. Analyzing Ctr2-mRNA and protein expression levels in six established human ovarian carcinoma cell lines of varying sensitivity to cisplatin it was shown that there was a significant correlation between Ctr2 expression at both the mRNA and protein levels and resistance to the cytotoxic effect of cisplatin. The higher the expression of hCtr2, the lower the observed sensitivity to cisplatin [93]. This suggests that Ctr2 expression may be one of the parameters that determine differences in cisplatin sensitivity in human ovarian carcinomas. Similar results have been obtained using carboplatin. Moreover, knockdown of Ctr2 was associated with an increase in cisplatin accumulation that was accompanied by an increase in cytotoxicity [93, 96], suggesting that selective inhibition of Ctr2 expression or function may be a useful strategy for enhancing the effectiveness of cisplatin chemotherapy. However, the role of Ctr2 in copper and cisplatin transport is not yet fully understood.

### 3.3. ATP7A and ATP7B

Other copper transporters that have been implicated in cisplatin cellular handling are the P-type copper-transporting ATPases ATP7A and ATP7B. Normally these transporters contribute to regulate the level of copper in cells, because excess copper is deleterious for cell metabolism. Inactivation of their transport activity is associated with diminished copper efflux from cells and, in some tissues, massive copper overload [57]. Harmful consequences of copper accumulation are particularly evident in the case of Wilson's disease, an autosomal recessive disorder caused by genetic inactivation of ATP7B. Patients affected by Wilson's disease have greatly elevated copper levels in the liver and in several other tissues and show a wide spectrum of hepatic abnormalities, including hepatitis, cirrhosis, fulminant liver failure, and/or neurological and psychiatric disease [57]. Conversely, specific mutations in the ATP7A gene cause Menkes' disease, a copper deficiency disorder, which is associated with impaired copper efflux from enterocytes into the blood and inadequate transport of copper to the brain. Patients with Menkes' disease show delayed growth and development, poor temperature control, connective tissue abnormalities, seizures, and mental retardation and die in their early childhood [57]. With regard to platinum compounds and specially cisplatin, while Ctr1 mediates platinum uptake into cells (see above), ATP7A and ATP7B seem to transport platinum out of cells or into specific subcellular compartments [97–102]. The first indication of the role of ATP7 transporters in determination of cisplatin cellular sensitivity comes from studies with human epidermoid carcinoma KB-3-1 cells [103]. Upon transfection with the ATP7B cDNA these cells showed an increased resistance to both cisplatin and copper, being the cellular accumulation and the efflux of cisplatin lower and higher, respectively, than in mock-transfected control cells [103]. ATP7B resulted to be overexpressed in cisplatin-resistant prostate carcinoma cells [103].

ATP7A is expressed in intestine and in choroid plexus, in vascular smooth muscle cells, vascular endothelial cells, and aorta as well as in cerebrovascular endothelial cells [104]. ATP7B is expressed in the liver and the brain [104]. Northern blot analyses demonstrated that the two copper ATPases are coexpressed in the brain and several other tissues, such as kidneys, lung, placenta, and mammary gland [104]. Three cisplatin-resistant human ovarian carcinoma cell lines exhibited increased expression of one or the other of ATP7A or ATP7B [105]. Further studies in human Menkes' disease fibroblasts that do not express either transporter and in sublines molecularly engineered to express either ATP7A or ATP7B demonstrated that these transporters influence the pharmacodynamics of cisplatin, carboplatin, and oxaliplatin [101]. The results were consistent with the hypothesis that these copper exporters sequester the platinum drugs into subcellular compartments, limiting their cytotoxicity, similar to their effect on copper. However, at least in this model system, although copper is readily exported after vesicular sequestration, the platinum drugs are not [101]. Comparing the expression of copper uptake and efflux transporters in three pairs of parent cell lines and cisplatin-resistant cell lines derived from various types of invasive oral squamous cell carcinoma, it was observed that ATP7B is correlated with the acquisition of cisplatin resistance more closely than either Ctr1 or ATP7A [106]. A further possible explanation of the development of cisplatin resistance was proposed studying the subcellular localization of ATP7A and ATP7B in sensitive and resistant cells by confocal fluorescence microscopy after immunohistochemical staining [107]. These experiments showed that, in the sensitive cells, both ATP7A and ATP7B transporters are mainly localized in the trans-Golgi network, whereas they are sequestrated in more peripherally located vesicles in the resistant cells. Changes in subcellular localization of ATP7A and ATP7B may facilitate sequestration of cisplatin in the vesicular structures of resistant cells, which may prevent drug binding to genomic DNA and thereby contribute to cisplatin resistance [107]. An important modulator of the ATP7A action on cisplatin is the Sec61 protein: this protein is able to transport proteins across lipid bilayers and regulate the expression and distribution of ATP7A [108].

Investigating the expression of ATP7A and ATP7B in ovarian cancer cell lines by real-time reverse transcription PCR and Western blot analysis, it was confirmed that ATP7A and ATP7B genes were expressed at higher levels in platinum-resistant cells compared with sensitive cells; however, only differences in ATP7B reached statistical significance. ATP7A gene silencing had no significant effect on the sensitivity of resistant cells to cisplatin, but ATP7B silencing resulted in 2.5-fold reduction of cisplatin sensitivity levels and increased DNA adduct formation in cisplatin-resistant cells [109]. Moreover, the therapeutic potential of ATP7B gene silencing for reversing platinum resistance was tested in vivo delivering ATP7B siRNA incorporated into neutral nanoliposomes into nude mice bearing tumors from the ovarian cancer cell line A2780-CP20. This approach in combination with cisplatin treatment was highly effective in reducing tumor growth. This effect resulted from reduced proliferation, increased tumor cell apoptosis, and reduced angiogenesis [109].

Finally, the expression of ATP7B in tumors seems to be related to cisplatin efficacy: expression of ATP7B correlates with cisplatin resistance in human non-small-cell lung cisplatin sensitivity of cancer xenografts [110]. Increased levels of ATP7B are associated with poor outcome in colorectal cancer patients receiving oxaliplatin-based chemotherapy [111].

Investigating the expression of ATP7A, ATP7B, and Ctr1 by real-time quantitative PCR, RT-PCR, immunohistochemistry, and Western blot analysis in DRG from healthy control adult rats or from animals treated with intraperitoneal oxaliplatin or drug vehicle, no expression of ATP7B in this tissue was observed [55]. Conversely, ATP7A was localized to the cytoplasm of cell bodies of smaller DRG neurons without staining of satellite cells and nerve fibres, while high levels of Ctr1 were detected in plasma membranes and vesicular cytoplasmic structures of the cell bodies of larger-sized DRG neurons without colocalization with ATP7A [55]. The authors of this study suggest that it is possible that ATP7A-expressing DRG neurons are less sensitive to oxaliplatin neurotoxicity because the high levels of ATP7A facilitate the cellular efflux of oxaliplatin reducing its availability for reactions with DNA or other key neurotoxicity targets. In contrast, DRG neurons expressing high levels of Ctr1 would be expected to take up more oxaliplatin leading to toxic effects in this neuronal subtype [55].

### 3.4. Organic Cation Transporters

First indications of the involvement of a renal transport system for cisplatin are derived from the finding that cisplatin is actively secreted in renal tubules [112]. The first direct evidence that organic cation transporters (OCTs) play a role in cisplatin transport is derived from experiments demonstrating that the uptake of tetraethylammonium (TEA, a model substrate for OCT) in NIH3T3 cells stably transfected with rat organic cation transporter 2 was competitively inhibited by cisplatin [113]. The vectorial nature of this transport was further confirmed in in vitro experiments with epithelial cells derived from proximal tubules of opossum (OK cell line), where the basolateral-to-apical transport of cisplatin was higher than apical-to-basolateral transport [114]. The involvement of OCT in the basolateral to apical cisplatin transport was suggested by coincubation experiments of cisplatin with TEA, which significantly decreased accumulation and transport of cisplatin from the basolateral to apical medium in OK cell line [114] and also in rabbit isolated proximal tubuli [115].

Other indications for the importance of OCTs in the uptake of cisplatin by proximal tubule cells come from studies with the clone C7 of Madin-Darby canine kidney (MDCK) cells. These cells have been demonstrated to express the subtype 2 of OCT and to be more sensitive to cisplatin toxicity when cisplatin was added to the basolateral versus luminal side [116]. This toxicity could be decreased by incubation with cimetidine, a substrate of OCT [116].

The function of OCT consists in mediating the transport of organic cations in or out of cells. Organic cations of both endogenous and exogenous origins are substrates of OCT. Endogenous organic cations are substances such as choline, dopamine, histamine, and creatinine, while among the exogenous organic cations we can find many drugs (up to 40% of prescribed drugs are organic cations [117]) such as vecuronium, procainamide, quinine, and cimetidine, only to give few examples. Evidently, these substances have a broad spectrum of molecular structures and dimensions that is the reason why OCTs are defined as polyspecific transporters. A common characteristic of OCT substrates seems to be positively charged at physiological pH.

OCTs are classified as uniporter belonging to the major facilitator superfamily (MFS). Organic cation transporters have been assigned to the SLC22A family that includes electrogenic organic cation transporters (OCT1-3), electroneutral organic cation transporters (OCTNs, OCTN1-3), and also a large group of transporters involved in organic anion transport (OATs, OAT1-5, and urate transporters, URAT1) [118, 119]. Since many of these transporters are expressed in intestine, liver, and kidney [119], the transporters of the SLC22A family play a pivotal role in drug absorption and excretion [119]. The nomenclature of transporters from other animal species follows the HUGO model, often with a lower case notation (e.g., slc22a1) to distinguish them from human transporters (e.g., SLC22A1).

Transport of organic cations mediated by the three OCT subtypes (OCT1, OCT2, and OCT3) is electrogenic, independent of Na^+^, and reversible with respect to direction [119]. The driving force is supplied solely by the electrochemical gradient of the transported organic cation. In this way, the uptake mediated by OCT is concentrative, in opposition to the transport mediated by Ctr1 that is equilibrative [5]. Therefore, OCTs can have a stronger activity than Ctr1 with respect to the transport of platinum derivatives [5]. There are only few data on the affinity of transporters for platin derivatives: Ctr1, for example, seems to have a *K*_*m*_ of 17 *μ*M for cisplatin [120], while the *K*_*m*_ of OCT2 for cisplatin transport was 11 *μ*M [121]. The physiology and pharmacology of organic cation transporters have been well summarized in different excellent reviews (see, e.g., [122–127]).

The cells of excretory organs, such as the kidney and the liver, have a morphological and functional polarization in order to support the vectorial movement of substances across cell membrane and across cells. This vectorial transport is mediated by the concerted action of transporters that are specifically expressed on the basolateral and apical plasma cell membrane [122, 124, 127, 128]. The first step for organic cation secretion in these organs is their absorption from the basolateral side into the cells. This process in human kidney is mainly mediated by OCT2, while in the liver it is supported by OCT1. The second step of the vectorial movement of organic cations is their secretion from the tubular cell into the tubular lumen in the kidney or from the hepatocytes into the bile in the liver. This process is mediated by different transporters: the P-glycoprotein (also named MDR1), an adenosine 5′-triphosphate (ATP) dependent transporter that probably mediates the efflux of bulky hydrophobic organic cations, and two H^+^/organic cation antiporters (OCTN1 and the mammalian proton cation antiporter called MATE1). According to their electrochemical gradient, organic cations can also be reabsorbed from the lumen into the interstitium. For this process, a polyspecific cation transport system mediating its uptake across the luminal membrane of proximal tubular cells has been proposed [128]. The efflux across the basolateral membrane into the interstitium may be mediated by OCT. The activity of these transporters can be acutely or chronically regulated.

Of special interest is the observation that the three OCT subtypes have a distinct and species-specific tissue distribution. Human OCT1 (hOCT1) is highly expressed in the liver on the sinusoidal membrane of hepatocytes [129, 130] and in jejunum (mainly on the lateral membranes of the enterocytes) [131]. hOCT2 was found to be preferentially expressed in the dopaminergic brain regions with the highest central expression in substantia nigra pars compacta [132] and in human kidney, where it is exclusively localized on the basolateral membrane of proximal tubule cells [133]. Conversely, the tissue expression pattern of OCT3 is very broad being expressed in brain, skeletal muscle, liver, placenta, and heart [127]. Interestingly, normal and tumor colon tissue samples express mRNA for hOCT1, while the mRNA for hOCT2 seems to be expressed at variable level only in colon cancer tissue samples [134]. PCR analysis of a panel of 18 permanent human tumor cell lines consisting of 6 different tumor types including four Ewing sarcoma cell lines (CADO-ES-1, STA-ET-1, STA-ET-2.1, and VH-64), four neuroblastoma cell lines (IMR5, KCN, SHEP-SF, and SH-SY5Y), two medulloblastoma cell lines (MNNG-HOS, UW228.2), two rhabdomyosarcoma cell lines (RD, RH30), two human acute lymphoblastic leukemia (ALL) T-cell lines (CCRF-CEM and MOLT-4), the human B-cell precursor ALL cell line REH, and the human acute myeloid leukemia cell line HL-60 showed that these cells do not express any OCT subtype. Importantly, these cells all express the human Ctr1 [89]. Conversely, in the renal cell carcinoma cell lines A498 and 7860, a highly significant expression of SLC22A3 (hOCT3) was detected by reverse transcription PCR and TaqMan real-time PCR [135]. It was also found that hOCT2 mRNA is expressed in ovarian cancer cell lines; however, its expression in clinical ovarian cancer specimens was low and did not correlate with the treatment outcome of platinum-based regimens [136]. Importantly, the tissue distribution of OCT is species-specific: for example, the subtype 1 is expressed in rodents both in the kidney and also in the liver [137, 138]. This fact should be taken into account when interpreting the results of translational studies.

In normal tissues, the OCTs apparently fulfill two main functions: on one hand they limit the action of aminergic neurotransmitters that have evaded high affinity uptake mechanisms [118] and, as in the case of hOCT2, they regulate the interplay between the endogenous neuromodulators of the central dopaminergic transmission [132]. On the other hand OCTs mediate the elimination of xenobiotics, thus representing an important system determining systemic exposition to drugs. Considering the specific tissue distribution of OCT (see above), this fact seems to be of special importance for determining specific effects and also side effects of drugs.

Several drugs have been demonstrated to be substrates or to interact with OCTs (e.g., the antihelmintic agent levamisole [139], the antiviral drugs 2′-deoxytubercidin [140, 141], acyclovir and ganciclovir [142], and lamivudine [143], *β*-adrenoceptor antagonists [144], nonsteroidal anti-inflammatory drugs [145], H2-receptor antagonists [146], antiarrhythmic drugs [147], and the serine-protease inhibitor nafamostat mesilate [148], the antihypertensive drug debrisoquine [149]—frequently used for phenotyping the drug metabolizing enzyme CYP2D6—the 5-hydroxytryptamine receptor 3 antagonists tropisetron and ondansetron [150], the antiepilectic drug lamotrigine [151], the chemotherapeutic drug ifosfamide [152], and the muscarinic antagonists oxybutynin and trospium [153]). In some cases, this interaction is important for the development of drug-induced toxicities, as demonstrated for pentamidine and furamidine [154], platinum derivatives [89, 134, 155–159], and biguanides [160–163].

In humans the genes coding for OCT1, OCT2, and OCT3 are localized within a cluster on chromosome 6.q26-7 [164–166]. Each of the three genes comprises 11 exons and 10 introns [165, 167, 168]. Interindividual variations in the pharmacokinetics of drugs may be caused by genetic variations in biotransformation and transmembrane transport [169]. Since membrane transporters influence drug absorption, distribution, and excretion and OCTs play an important role in the metabolism of many medications, polymorphisms of OCT can be of relevance in determining how patients respond to pharmacological therapy or how they are predisposed to develop drug adverse effects [2]. Indeed, the genetic variants of OCT1 P283L and P341L and of OCT2 T199I, T201 M, and A270S, which were identified in a Korean population, decreased the transport of lamivudine in vitro [170]. Another example of the pharmacological importance of OCT genetic variation is furnished by the demonstration that the presence of the hOCT2 polymorphism A270S significantly changes the renal clearance and the net secretion of metformin in healthy volunteers [171].

Focusing on cisplatin, the first direct demonstration of cisplatin transport by OCT came from in vitro studies with HEK293 cells stably transfected with hOCT. In this system, cisplatin showed a significant interaction with organic cation transport mediated by hOCT2 but not with that by hOCT1. In human proximal tubules cisplatin competed with basolateral organic cation transport, whereas it did not influence organic cation transport in hepatocytes. Incubation of hOCT2 expressing cells with cisplatin induced apoptosis, which was completely suppressed by contemporaneous incubation with the hOCT2 substrate cimetidine [155]. These findings demonstrated that uptake of cisplatin is mediated by hOCT2 in renal proximal tubules, furnishing an explanation for its organ-specific toxicity. Moreover, they suggested that a combination of cisplatin with other substrates that compete for hOCT2 may offer an effective option to decrease nephrotoxicity in the clinical setting. Contemporaneously, it was demonstrated that HEK293 cells stably expressing rat OCT2 (rOCT2) were significantly more sensitive to cisplatin toxicity than cells not expressing the transporter [157]. Cimetidine and corticosterone, both OCT2 inhibitors, decreased the cytotoxicity and the transport of cisplatin by rOCT2. Investigating the pharmacokinetics of cisplatin in male and female rats, it was found that the renal uptake clearance of cisplatin was greater in male than female rats, while the hepatic uptake clearance was similar between the sexes [157]. Also the cisplatin renal toxicity was more severe in male than female animals [157]. Apparently, this is due to stronger expression of rOCT2 in renal proximal tubules from male compared with female animals [157, 172]. The implication of OCT in cisplatin transport and toxicity was further confirmed in studies, where it was demonstrated that the OCT2-mediated transport of cisplatin was saturable and that cisplatin inhibited OCT2-mediated transport of tetraethylammonium (TEA) by up to 97% [121]. Other studies showed that hOCT2 mediates not only cisplatin uptake and toxicity, but has even a stronger interaction with oxaliplatin [134, 136]. The above-mentioned studies were performed in in vitro systems. In vivo the importance of OCT transporters in mediating cisplatin toxicities has been demonstrated in mice, where OCT1 and OCT2 were genetically deleted. Deletion of OCT1 and OCT2 resulted in significantly impaired urinary excretion of cisplatin without an apparent influence on its plasma levels [173]. Furthermore, the OCT1/OCT2-deficient mice were protected from severe cisplatin-induced renal tubular damage. These findings were confirmed in another study with the OCT1/OCT2-deficient mice, where it was demonstrated that cisplatin nephrotoxicity is milder in these mice compared with wild-type animals [89]. In this study, for the first time it could be demonstrated that OCT2 is also expressed in the cochlea in the outer hair cells and also in cells of the stria vascularis and that genetic deletion of OCT1 and OCT2 protected the mice also from cisplatin ototoxicity. Treatment of the wild-type animals with cisplatin together with cimetidine, a competitor for the transport by OCT, eliminated or lowered the ototoxic and nephrotoxic effects of cisplatin, respectively. Very importantly, in another animal study, it could be shown that treatment of rats with cisplatin together with cimetidine did not interfere with the antitumoral activity of cisplatin [174]. Collectively, these results indicate the critical importance of OCT2 in the handling in the kidney and in the ear and related toxicity of cisplatin and provide a rationale for the development of new targeted approaches to mitigate these debilitating side effects of cisplatin chemotherapy.

Cisplatin treatment causes disturbances in renal handling of electrolytes. In particular, hypomagnesemia has emerged as a common event associated with cisplatin therapy [175, 176]. Investigating the mechanisms of cisplatin nephrotoxicity, it was shown that rats fed a magnesium-deficient diet showed a significant body weight decrease and a marked decrease in serum magnesium levels compared with control rats fed on adequate magnesium diet [177]. Hypomagnesemic rats were more sensitive to cisplatin nephrotoxicity than control rats [177]. Immunoblotting and uptake experiments revealed specific upregulation of the expression and function of the organic cation transporter 2 in hypomagnesemic rats before cisplatin administration, accompanied by an increase in renal accumulation of cisplatin. The authors suggest that hypomagnesemia could cause dehydration and upregulation of rOCT2, enhancing renal accumulation of cisplatin and the deterioration of renal function [177].

The role of genetic variation of hOCT2 in determining pharmacokinetics and nephrotoxicity of cisplatin is under debate: screening the DNA of 106 cancer patients for genetic variation, only one single nucleotide polymorphism (A270S; rs316019) was identified (minor allele frequency, 7.6%). This polymorphism did not appear to be associated with any of the studied pharmacokinetic variables [121]. In another study, it was found that the same nonsynonymous single-nucleotide polymorphism in the OCT2 gene was associated with reduced cisplatin-induced nephrotoxicity in patients [173]. On the contrary, screening the DNA of 79 cancer patients receiving cisplatin-containing chemotherapy showed that polymorphisms in hOCT2 genes were not associated with cisplatin-induced nephrotoxicity [178]. Studying a total of 53 patients with advanced carcinomas, it was demonstrated that cancer patients carrying the OCT2 (A270S) genotype were less susceptible to cisplatin-induced nephrotoxicity, but not to hematological toxicity [179].

Recently, also hOCT3 has been involved in the transport of cisplatin because the cisplatin-sensitive cervical adenocarcinoma KB-3-1 cells express extremely higher level of hOCT3 than its derivative cisplatin-resistant KB-CP20 cells. OCT3 overexpression significantly increased cisplatin cellular accumulation and cytotoxicity in KB-3-1 cells, while its downregulation by short hairpin RNA or chemical inhibition increased their resistance [180]. Intriguingly, there was no effect of OCT3 overexpression on cisplatin accumulation and cytotoxicity in human embryonic kidney 293 cells, suggesting that the role of OCT3 in transport of cisplatin and determination of its cytotoxicity in in vitro experiments might be dependent on the cell type under study. Indeed, other authors failed to identify a transport of cisplatin by OCT3, at the same time showing a role for OCT3 in oxaliplatin uptake [158]. Moreover, it was also found that the level of hOCT3 mRNA in the colon was 9.7-fold higher in cancerous than in normal tissues in six Japanese patients and that in human colorectal cancer-derived cell lines, the mRNA of hOCT3 was highly expressed compared with that of other organic cation transporters [181].

Interestingly, studies with mice, where the OCT1 and OCT2 were genetically deleted (OCT1/2^(−/−)^ mice), showed that cisplatin caused an increase in the urinary excretion of carnitine (the substrate of OCTN2) and acetylcarnitine in wild-type mice but not in OCT1/2^(−/−)^ mice, suggesting a pivotal role of OCT1 and OCT2 in cisplatin-related disturbances in carnitine homeostasis [182]. In this study it has been shown that cisplatin did not directly inhibit the transport of carnitine by OCTN2 but downregulated multiple target genes of the transcription factor peroxisome proliferator-activated receptor *α*, including Slc22a5, in the kidney of wild-type mice. These effects were absent in OCT1/2^(−/−)^ mice. The mechanism by which cisplatin causes deregulation of OCTN2 expression in the kidney of wild-type mice is not entirely clear. The authors of this work proposed an explanation starting from the consideration that the expression of mouse OCTN2 is directly governed by the transcription factor peroxisome proliferator-activated receptor *α* (Ppar-*α*) and that this process is mediated through a functional peroxisome proliferator response element located in the first intron [182]. Following cisplatin administration, the drug is taken up into renal tubular cells by OCT1 and OCT2, with perhaps a minor role of the copper transporter Ctr1, where the interaction between cisplatin and Cyp2e1 results in the generation of reactive oxygen species. The accumulation of reactive oxygen species then triggers, directly or indirectly, the activation of Mapk14 (the p38 mitogen-activated protein kinase), leading to increased production of tumor necrosis factor-*α*. As a result, the tubular expression of multiple cytokines, including IL-1*β*, is increased, and this subsequently causes a decrease in the expression of Pargc-1*α*, a tissue-specific transcriptional coactivator of Ppar-*α* and its obligate partner retinoid X receptor. The reduced availability of Pargc-1*α*, along with the ability of cisplatin to directly impair DNA-binding activity to the Ppar-*α*/retinoid X receptor-*α* heterodimer, decreases the expression of Ppar-*α* targets such as OCTN2 and ultimately leads to reduced reabsorptive capacity and loss of carnitines in urine. This proposed mechanism well illustrates the possible complicated consequences of renal cisplatin uptake by OCT.

### 3.5. Multidrug and Toxin Extrusion (MATE) Transporters

Another transporter that has been described to be able to interact with cisplatin is the multidrug and toxin extrusion 1 (MATE1) transporter. Two MATE transporters were identified (MATE1 and MATE2, SLC47A1 and SLC47A2, resp.) and were assigned to the SLC47 family [183]. Human MATE2 was cloned as a homologue of human MATE1 [184]. Two splice variants of MATE2 have been described. The variant MATE2-K is predominantly expressed in human kidney and is the active form of the SLC47A2 gene [185]. Animal orthologues of the human MATE have also been found: the characteristics of human MATE1 and rodent MATE1 are similar, but the counterparts of human MATE2-K have not been identified in rats and mice, and the counterpart of rodent MATE2 has not been found in humans [183]. Functional characterization of MATE showed that they are H^+^/organic cation antiporters [186]. Human MATE1 is highly expressed in the kidney, adrenal gland, liver, skeletal muscle, and several other tissues [185]. MATE2-K exhibits a kidney-specific expression. MATE1 and MATE2-K mRNA are detected at similar levels in the kidney, and the proteins are localized in the brush-border membrane of proximal tubules. Therefore, both MATE1 and MATE2-K could play a role in the renal tubular secretion of cationic drugs in human. MATE1 also acts as an efflux transporter in other tissues. Species differences in the tissue distribution of organic cation transporters were demonstrated. The tissue distribution of MATE1 in mice is generally consistent with that in human. However, MATE2-K is not expressed in mice. In the case of MATE, MATE1 knockout mice could represent a model of MATE1 and MATE2-K deficiency in humans [183, 187]. Typical organic cations are substrates for MATE1 and MATE2-K [188]. Most of these compounds are also transported by OCT2 [183]. *K*_*m*_ values of cationic drugs for MATE1 and MATE2-K are similar and higher than the plasma concentrations in clinical use. Moreover, MATE1 can transport some organic anions that are substrates of organic anion transporters. With respect to substrate specificity, MATE1 and MATE2-K are very similar, but not completely the same [183].

In a first study investigating the uptake of cisplatin by hMATE1 and hMATE2 K transfected in HEK293 cells, the cellular uptake of cisplatin for 1 h was increased by the expression of hMATE1 and hMATE2-K without pretreatment with ammonium chloride [158]. Comparing the uptake of cisplatin, carboplatin, nedaplatin, and oxaliplatin in cells transfected with the rat MATE1 (rMATE1) under conditions of ammonium chloride-generated intracellular acidification, it was observed that only the uptake of oxaliplatin was significantly increased by the expression of rMATE1 [156]. Pretreatment with ammonium chloride was performed because the MATE transporters are activated by the oppositely generated H^+^-gradient across the plasma membrane. Extending the experiments to HEK293 cells expressing hMATE1 and hMATE2-K, it was found that the expression of hMATE2-K and also hMATE1 significantly stimulated the H^+^-gradient-dependent uptake of oxaliplatin. In these cells there was no significant stimulation of the accumulation of cisplatin, carboplatin, and nedaplatin [156]. Even though the transport of platinum derivatives by MATE is not clearly demonstrated, experiments with mice, where the mouse MATE1 (mMATE1) was genetically deleted (MATE1(−/−)), showed that the nephrotoxicity of cisplatin was potentiated in MATE1(−/−) mice compared with wild-type (Mate1(+/+)) mice [189]. Moreover, the renal accumulation of cisplatin was significantly increased in Mate1(−/−) mice. On the other hand, MATE1 was only found to play only a minor role in the hepatic accumulation and hepatotoxicity of cisplatin [189]. The authors of this study proposed that MATE1 is involved in cisplatin-induced nephrotoxicity because it mediates the efflux of cisplatin from renal tubular epithelial cells and that genetic deficiency or drug drug interaction in MATE would potentiate the nephrotoxicity in cisplatin-based chemotherapy [189]. Considering that the cells of the renal proximal tubules possess a functional and morphological polarisation to exert vectorial transport processes, it could be speculated that the uptake of cisplatin from the basolateral side happens through the action of OCT2 and Ctr1; once cisplatin accumulates in these cells, its transport into the urine will be mediated at the apical side by MATE transporters.

Indeed, when cimetidine was used to protect wild-type mice from cisplatin toxicity, only a mild protective effect was observed in the kidney, while the ototoxicity was completely suppressed [89]. Since cimetidine has a higher affinity for MATE1 than for OCT2 [190], a possible explanation of this finding could be that cimetidine inhibits the efflux of cisplatin by MATE1 and less effectively its uptake in renal tubular proximal cells by OCT2, resulting only in a mild protection from cisplatin toxicity.

In the SLC47A1 and SLC47A2 genes, 11 and 2 nonsynonymous SNPs, respectively, were found, some of which affected the transporter function. The mutations G64D and V480 M in MATE1 and G211V in MATE2-K caused a complete loss of function [191, 192], in this way affecting the pharmacokinetics of all substrates. In addition, polymorphisms in the promoter region of MATE1 with effects on binding and transcription activity of transcription factors were identified [193]. In a population-based cohort study in diabetic patients it was observed that SNP rs2289669 G>A in MATE1 is associated with the glucose-lowering effect of metformin, a substrate of this transporter [194]. This finding is consistent with a reduction in MATE1 transport activity, that is, extrusion of cellular drugs from renal tubular cells into the lumen. However, screening the DNA of 53 patients with advanced carcinomas, this SNP resulted not to be associated with the adverse effects of cisplatin, such as nephrotoxicity [179]. It was suggested that the MATE1 genetic variant could not affect the apical efflux transport of cisplatin out of the renal cells, thereby not being involved directly in the development of nephrotoxicity [179].

On the other hand, oxaliplatin was a specific substrate for MATE2-K, although other platinum agents were hardly recognized by MATE2-K [156, 158].

Investigating the expression of SLC22 family transporters in rat DRG, rOCT2, rOCT2, rOCTN1, and rOCTN2 were detected by real-time PCR and Western blot analysis [195]. Measuring the [^14^C] oxaliplatin uptake and oxaliplatin-induced cytotoxicity and their inhibition by specific substrates of OCTN1 and 2 (ergothioneine and l-carnitine, resp.) in HEK293 cells overexpressing the rat and human genes (rOCTN1, rOCTN2, hOCTN1, and hOCTN2) relative to mock-transfected control cells, the authors demonstrated that oxaliplatin is transported by rat and human OCTN1 and OCTN2. DRG tissue and primary cultures from female Wistar rats displayed an oxaliplatin uptake, whose characteristics resemble that of an OCTN1-mediated transport [195]. It should be noted that these studies have been performed using tissues from female rats. It is known that female rats have a much lower expression of OCT2 compared with male rats and other rodents [157, 196, 197], suggesting that these findings underestimate the possible role of OCT2 in the development of neurotoxicity. Therefore, the relative contributions of copper transporters, OCTs, and OCTNs to the neurotoxicity of platinum derivatives remain to be determined definitively.

## 4. Concluding Remarks

At the end of this paper, a few words should be spent on the cellular uptake processes for other platinum derivatives, which has been developed in the attempt to obtain platinating agents that have a more convenient route of administration and a lower toxicity profile than cisplatin, at the same time displaying no cross-resistance with cisplatin, and activity also in platinum-nonresponsive cancers.

Nedaplatin, a second-generation cisplatin analogue with antineoplastic activity, appears to be less nephrotoxic and neurotoxic compared to both cisplatin and carboplatin. Nedaplatin was shown not to be transported by rOCT1-3 and rMATE1 [156], hOCT1-3, hOCTN1, hOCTN2, hMATE1, and hMATE2-K [158].

Satraplatin is the first orally administered platinum drug under active clinical investigation in patients with prostate cancer [198]. Satraplatin and its major metabolite, cis-ammine dichloro(cyclohexylamine) platinum(II) (JM118), have been demonstrated to have antitumor effects in vitro, in vivo, and in clinical trials. Since satraplatin is lipophilic, its cellular accumulation should be independent on specific membrane transporters [199]. However, the transport of JM118 seems to be mediated by OCT, at least in cancer cell lines [200]. ATP7A and ATP7B have been demonstrated to mediate resistance to JM118, and it has been suggested that they sequester JM118 into vesicular compartments within the cell resulting in enhanced whole cell accumulation and reduced cytotoxicity [201]. On the other hand, JM118 seems not to interact with Ctr1 [201].

Picoplatin is a new-generation platinum compound, which has displayed a spectrum of activity distinct from its predecessors in addition to an improved safety profile. To date, it has shown no indication of significant nephrotoxicity, ototoxicity, or neurotoxicity and promising activity in platinum-sensitive, platinum-resistant, and refractory disease [159]. Picoplatin seems to form unique DNA adducts, which helps it to retain its anticancer activity also in cells that have acquired cisplatin resistance [202]. Since picoplatin is proposed for the treatment of lung cancers, the expression of OCT1 and OCT2 was investigated in normal and tumor lung tissue. Both transporters were detected in this tissue, with the expression of OCT2 being the lowest [159]. Picoplatin seems to be a substrate of OCT1 and OCT2, and their expression correlates with picoplatin toxicity. On the other hand, Ctr1, MATE1, and MATE2 K failed to show any significant contribution to picoplatin cytotoxicity [159]. However, in vivo pharmacokinetic experiments in mice with genetic deletion of OCT1 and OCT2 did not reveal any statistically significant differences in the pharmacokinetic profiles of picoplatin even though knockout mice exhibited a trend toward higher mean plasma concentration when compared with the wild-type mice [159].

In conclusion, membrane transporters play an important role for the cellular uptake of platinating agents such as cisplatin and oxaliplatin. Some transporters like Ctr1 and ATP7B seem to be involved in determining general sensitivity of tumor cells against platinum-based chemotherapy. The OCT2 may be also involved in the therapeutic uptake of oxaliplatin in intestinal cells. However, these transporters are also involved in determining undesired side effects such as nephrotoxicity (Ctr1, OCT2, and MATE), ototoxicity (Ctr1 and OCT2), and neurotoxicity (Ctr1, OCTN1). The identification of SNPs in these transporters and of their importance in determining effectiveness and toxic effects of drug treatment opened the field to personalized medicine, which promises a path for individually optimized treatment choices [203]. Moreover, the exact knowledge of the mechanisms governing the uptake of platinating agents in target and also nontarget cells is mandatory to establish specific protective treatment protocols.
